# Palatal petechiae: an uncommon oral adverse effect of COVID-19 vaccine

**DOI:** 10.1186/s43163-021-00167-w

**Published:** 2021-09-25

**Authors:** Bhawna Sayare, Vinay Kumar Bhardwaj, Deepak Sharma

**Affiliations:** 1 Department of Public Health Dentistry, HP Government Dental College and Hospital, Shimla, Himachal Pradesh India; 2 Department of Periodontology, HP Government Dental College and Hospital, Shimla, Himachal Pradesh India

**Keywords:** Adverse effect, COVISHIELD, ChAdOx-nCoV-19, Petechiae

## Abstract

**Background:**

The authors present an unusual, unreported oral adverse effect of COVID-19 vaccine as palatal petechiae lesions.

**Case presentation:**

COVID-19 disease caused by severe acute respiratory syndrome coronavirus 2 has become a global health crisis and has caused millions of deaths worldwide. Vaccination programs have been initiated in many countries for COVID-19 prevention. The ChAdOx-nCoV-19 vaccine has been shown to provide a robust immune response in various clinical trials. It is well tolerated by recipients and has been associated with minor adverse effects. The COVISHIELD (ChAdOx-nCoV-19) vaccine developed by AstraZeneca/Oxford University is approved by the Government of India to be administered in a phased manner. We present the case of twin subjects who suffered various adverse effects after receiving the COVISHIELD vaccine; one of the twins presented with palatal petechiae lesions presumably caused by the vaccine. The lesions were self-limiting and required no treatment.

**Conclusion:**

Oral lesions reported first time in our case should be correlated with similar lesions found post-COVID vaccines globally. There is a further need to ascertain a high level of scientific evidence and explore the biological plausibility underlying oral complications and COVID vaccinations.

## Background

WHO has reported 105,805,951 confirmed cases of COVID-19, including 2,312,278 deaths, globally as of 8 February 2021. In India, there have been 10,838,194 confirmed cases of COVID-19 with 155,080 deaths [1]. To limit the COVID-19 pandemic, an effective and safe vaccine against severe acute respiratory syndrome coronavirus 2 (SARS-CoV-2) will prove very instrumental. Vaccines based on various platforms, namely lipid nano-particle m-RNA, DNA, adjuvant protein, inactivated virus particles, and non-replicating viral vectors, are currently in different phases of clinical trials. ChAdOx1-nCoV-19 is a replication-defective chimpanzee adenovirus-vectored vaccine expressing the full-length SARS-CoV-2 spike glycoprotein gene. It provides a robust humoral and cellular immune response against the virus and is found to be well tolerated with minimum adverse effects [2].

Serum Institute of India, Pune, has presented the COVISHIELD vaccine with technology transfer from AstraZeneca/Oxford University [3]. COVISHIELD, a recombinant ChAdOx1-nCoV-19 vaccine, is approved by the Government of India as a vaccine against SARS-CoV-2 as a 2 dose regime. It is administered as a single intramuscular injection into the deltoid according to the standard operating procedures. The different adverse effects of the vaccine include the common ones such as nausea, vomiting, headache, myalgia, arthralgia, tenderness and pain at the injection site, fatigue, malaise, and pyrexia. Uncommon adverse effects include lymphadenopathy, rash, decreased appetite, and abdominal pain.

The authors report for the first time an uncommon oral adverse effect as petechiae lesions on the palate presumably caused by the vaccine in one of the twin subjects who received COVISHEILD (ChAdOx1-nCoV-19) vaccine.

## Case presentation

**Fig. 1:**
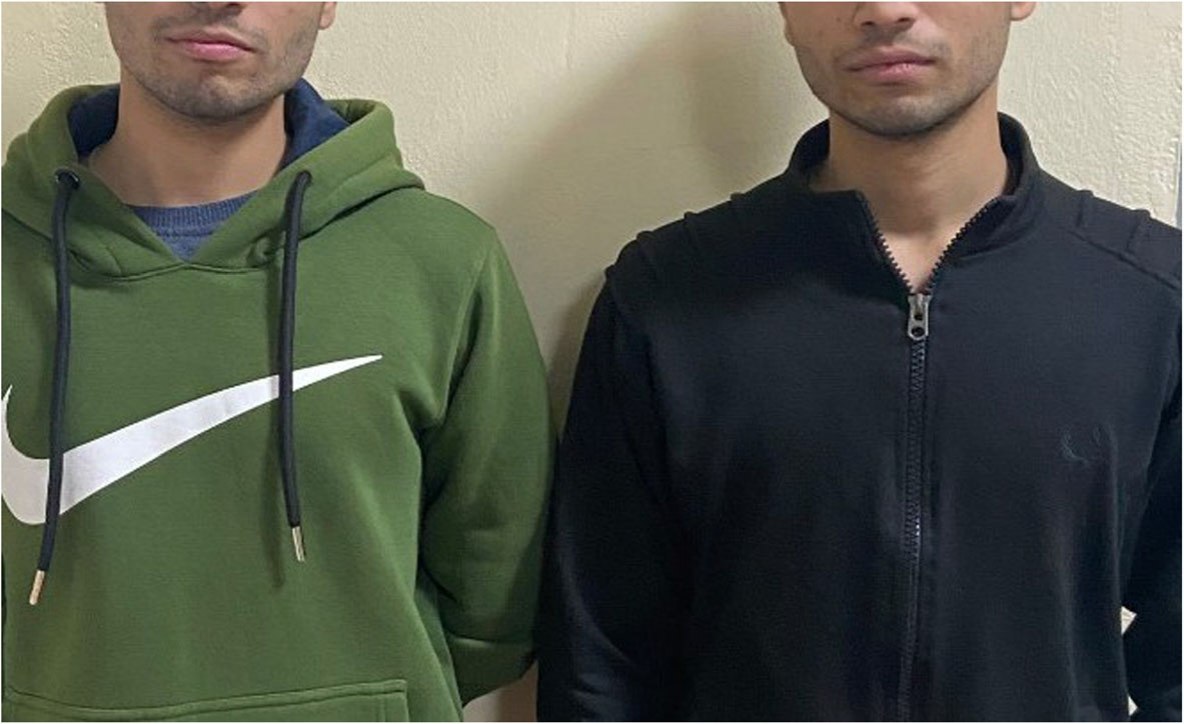
Twin subjects with adverse effects of COVID vaccine

**Fig. 2:**
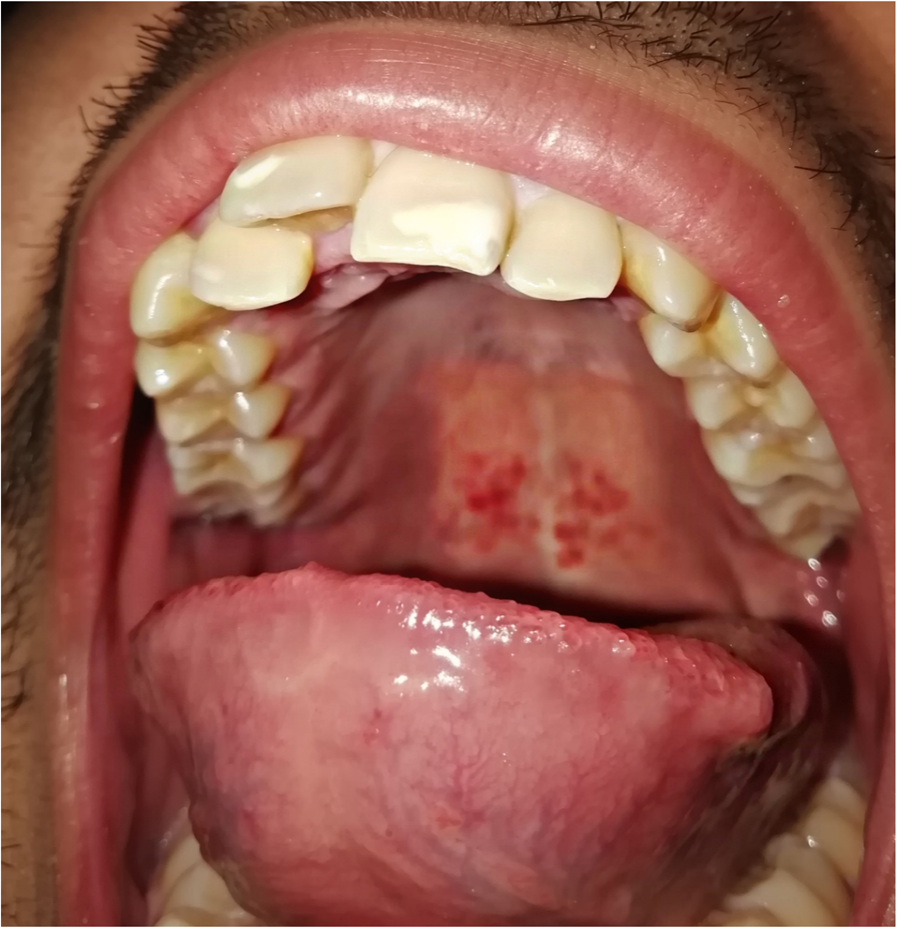
Petechiae lesions on the palate post-COVID vaccine

**Fig. 3:**
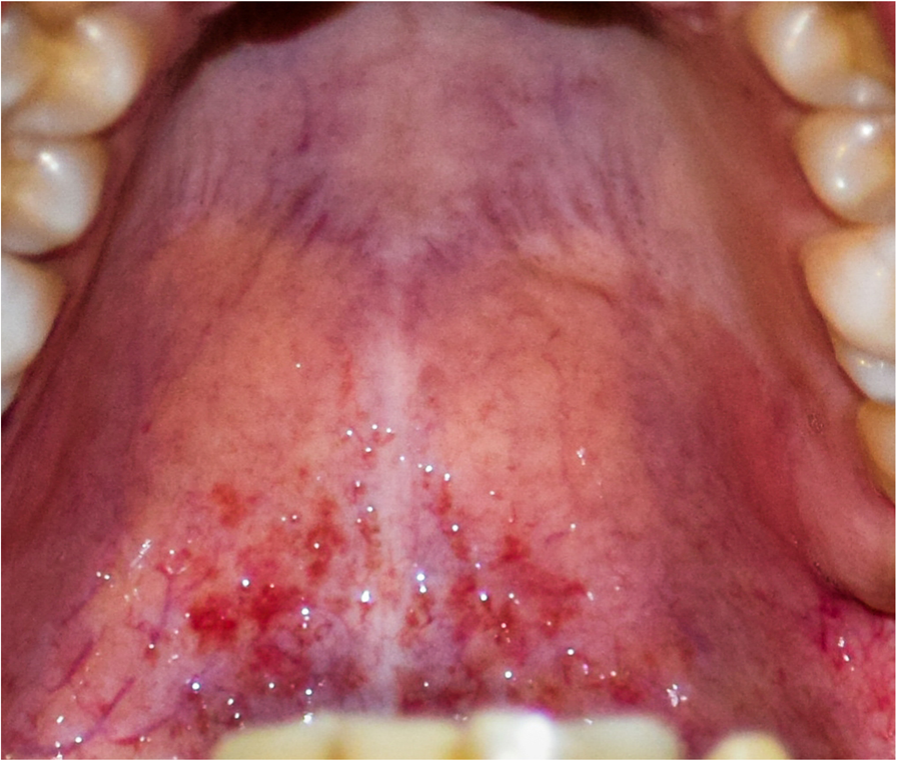
Reduced lesions on the third day

**Fig. 4:**
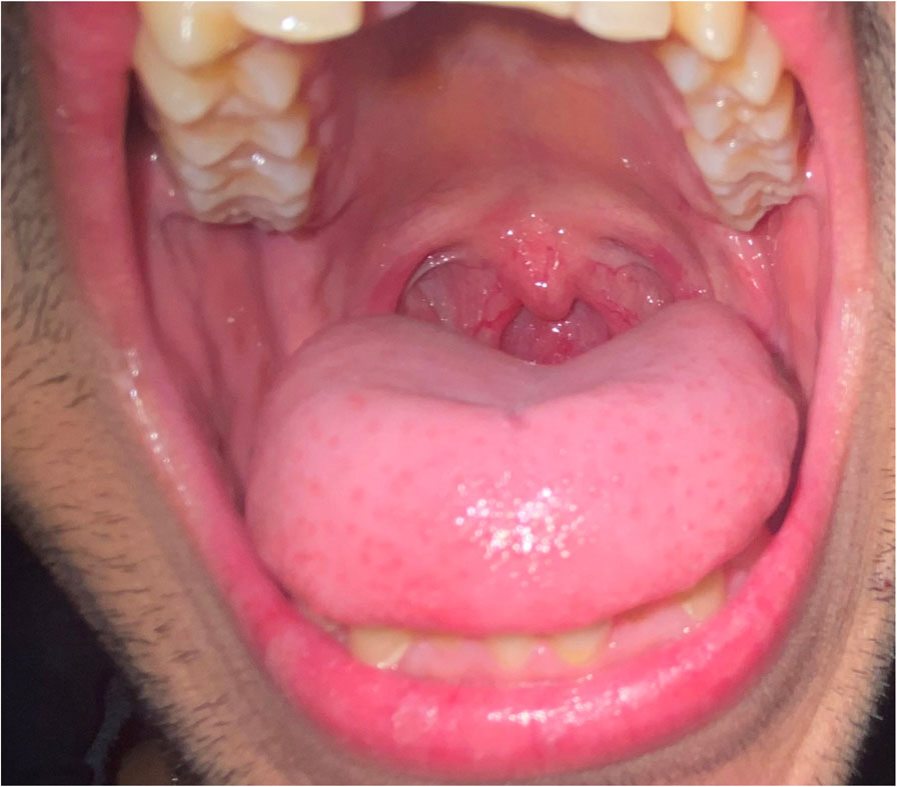
Completely healed lesions on the fifth day

The twin subjects aged 21 years, otherwise systemically healthy with no history of anticoagulants, anti-platelets, or other drugs intake (Fig. 1), received the first dose of COVISHIELD (ChAdOx1-nCoV-19) vaccine in the morning hours on day 1. After 4 h, the subjects complained of shivering, fever (102 °F), pain and tenderness at the site of injection, dry mouth, loss of smell, loss of appetite, difficulty in swallowing, cough, and severe headache in the occipital region. The subjects did not have any previous history of allergy or any significant medical or dental condition. One of the twin subjects experienced petechiae at the site of injection and in the palatal region (Fig. 2). General symptoms persisted for 3 days and were relieved thereafter. Petechiae on the palate also decreased on the third day and subsided completely on the fifth day (Figs. 3 and 4). The patient’s platelet count was reduced to 140,000 platelets per microliter of blood on the third day and rose to 225,000 on the tenth day without medical treatment. The petechiae lesions were self-limiting in nature and required no active treatment. Subjects took paracetamol for the first 3 days of vaccination.

SARS-CoV-2 infection may cause a myriad of systemic complications such as respiratory, cardiac, renal, gastrointestinal, and neurological complications. SARS-CoV-2 binds to angiotensin-converting enzyme 2 (ACE-2) receptors found in many organs and tissue including epithelial cells of the tongue and salivary glands. Evidences suggest a role of the oral cavity and oral mucosa in the transmission and in the pathogenicity of SARS-CoV-2 [4]. Oral lesions like ulcerations and necrosis have been found in patients diagnosed with SARS-CoV-2 infection [5]. The pathophysiology of oral lesions in SARS-COV-2 infection remains unclear. As the COVID-19 pandemic is a global crisis, everybody is hoping for an early vaccine. Various vaccines have been approved in different countries against the SARS-CoV-2 virus and are associated with various adverse effects. COVISHIELD being a version of the Oxford University-AstraZeneca viral vector vaccine, ChAdOx1 nCoV-19 is found to be efficacious and could contribute to control of this pandemic [6].

Venous thrombosis and thrombocytopenia after ChAdOx1 nCoV-19 vaccine administration is reported previously by Schultz et al. The affected patients had high levels of antibodies to platelet factor 4–polyanion complexes [7]. To the authors’ best knowledge, this is the first reported case of oral complication of the COVISHIELD vaccine. The petechiae lesions were self-limiting and required no treatment.

## Conclusions

Vaccines have their own safety concerns. The rapid development of the COVID-19 vaccine is in need of the hour, but rigorous long-term research with an adequate sample population is required to determine the vaccine safety. Oral lesions reported first time in our case should be correlated with similar lesions found post-COVID vaccines globally. There is a further need to ascertain a high level of scientific evidence and explore the biological plausibility underlying oral complications and COVID vaccinations.

## Data Availability

Available
